# Control Effect and Possible Mechanism of the Natural Compound Phenazine-1-Carboxamide against *Botrytis cinerea*

**DOI:** 10.1371/journal.pone.0140380

**Published:** 2015-10-13

**Authors:** Ya Zhang, Chong Wang, Pin Su, Xiaolan Liao

**Affiliations:** 1 Department of Plant Protection, College of Plant Protection, Hunan Agricultural University, Changsha, P. R. China; 2 Department of Chemistry, Science College, Hunan Agricultural University, Changsha, P. R. China; 3 Laboratory of Biocontrol, Institute of Plant Protection, Hunan Academy of Agricultural Sciences, Changsha, P. R. China; 4 Hunan Provincial Key Laboratory for the Biology and Control of Plant Diseases and Plant Pests, Changsha, P. R. China; University of California, UNITED STATES

## Abstract

To develop new agents against strawberry grey mould and to aid in the development of biological pesticides, we investigated the inhibitory effect of a natural compound, phenazine-1-carboxamide (PCN), against *Botrytis cinerea* using a growth rate assay. Additionally, indoor toxicity and the *in vitro* control effect of PCN were further studied to determine its potential mechanisms of action on *B*. *cinerea*. PCN was inhibitory against *B*. *cinerea* with a 50% effective concentration (EC_50_) of 108.12 μg/mL; the toxicity of PCN was equivalent to that of carbendazim (CBM). The best *in vitro* control effect of PCN against grey mould in strawberry (fruit) reached 75.32%, which was slightly higher than that of CBM. The field control effect of PCN against grey mould reached a maximum of 72.31% at a PCN concentration of 700 μg/mL, which was 1.02 times higher than that of CBM. Fungistatic activity was observed at low concentrations of PCN, while high concentrations of PCN resulted in fungicidal activity against *B*. *cinerea*. This natural compound strongly inhibited both spore and sclerotium germination of *B*. *cinerea*, with the best relative inhibition rates of 77.03% and 82.11%, respectively. The inhibitory effect of PCN on mycelial growth of *B*. *cinerea* was significant and reached levels of 87.32%. Scanning electron microscopy observations revealed that after 48 h of PCN treatment, the mycelia appeared loose, locally twisted, and folded, with exudation of contents; the mycelia was withered and twisted, with edge burrs, deformations, ruptures and a sheet-like structure. Transmission electron microscopy observations revealed that after 48 h of PCN treatment, the structure of the cell nucleus was unclear and the vacuoles had ruptured; additionally, various organelles exhibited disordered structures, there were substantial non-membrane transparent inclusions, the cells were plasmolysed, the cell walls were collapsed in some cases, and the hyphal tissue was essentially necrotic. A PCN dosage of 35–140 μg/mL had no effect on the cell membrane permeability of the mycelia, while a PCN dosage of 700 μg/mL resulted in significant permeability. PCN inhibited *B*. *cinerea* toxin; the mycotoxin level was approximately 0.41 of the value recorded for the control at a PCN dosage of 700 μg/mL. PCN affected the activity of pectin methylgalacturonase (PMG), polygalacturonase (PG), cellulase (Cx) and *β-*glucosidase (BG); the lowest activities of PMG, PG, BG and Cx reached 0.3 U/mg, 0.62 U/mg, 0.64 U/mg, and 0.79 U/mg, respectively, after treatment with 700 μg/mL PCN.

## Introduction

Strawberry is a perennial fruit of the genus *Fragaria*, family Rosaceae. After it is planted, strawberry can be harvested multiple times. This fruit has a high yield and nutrient concentration, and good economic benefits [1]. In recent years, the strawberry industry has developed rapidly as a result of increased consumption of this fruit. Strawberry cultivation has high technical requirements, and the fruit is inconvenient to transfer. Together with continuous cropping and other factors, the accumulation of plant pathogens often occurs, leading to more severe occurrence of diseases [2,3]. Strawberry grey mould is an important disease of strawberry from flowering to the fruiting stage, and is also the main factor affecting strawberry yield and quality [4–6]. Strawberry grey mould is a fungal disease caused by *Botrytis cinerea*. Control of this disease is very difficult because of the extensive host range of more than 200 plant species; *B*. *cinerea* infects the bud, flower, calyx, fruit, and leaf through spores or mycelia. Under low temperatures and high humidity, the occurrence of strawberry grey mould can be extremely severe, with 10–20% annual losses in yield [7–9].

Presently, the main methods to control strawberry grey mould include planting disease-resistant cultivars [10], changing cultivation methods [11], chemical control [12], and biological control [1]. Thus far, cultivars highly resistant to strawberry grey mould have not been identified, making it difficult to implement breeding for disease resistance [10]. Changing cultivation methods requires complete facilities to control field conditions, which is difficult to realise under the existing conditions in China [3]. In practical production, the control of strawberry grey mould primarily relies on chemical agents. Commonly used agents include benzimidazole, dicarboximide and N-phenylcarbamate fungicides. However, the long-term and extensive use of chemicals pollutes the environment and destroys the ecological balance; in addition, their use readily results in drug resistance in *B*. *cinerea*, which reduces the control effect [13–16]. More recently, with the improvement of people’s living standards and the enhancement of food safety awareness, finding new methods or alternatives to chemical pesticides to control strawberry grey mould has become a hot research topic. Biological control of strawberry grey mould has the advantages of high efficiency, low toxicity, environmental protection and safety, and thus has become a focus of relevant research. ‘Using microorganisms to control pathogens’ is one of the main aspects of biological control [17–19]. To date, the biocontrol agents used to control strawberry grey mould mainly consist of fungi [20,21] and bacteria [1,22], of which bacteria are an important resource; the reported species include members of *Bacillus*, *Xanthomonas*, *Pseudomonas*, and *Erwinia* [23,24]. The most common biocontrol bacteria are *Bacillus subtilis*, *Brevibacillus brevis*, and *Pseudomonas fluorescens* [25,26]. However, although different research teams have screened various biocontrol agents with specific activities, the progress of most studies is slow, and many agents remain at the laboratory stage. The possible reason for this limitation is related to the easy degeneration of the strains, short-lasting periods, slow control effects and the influence of environmental conditions. In our preliminary study, an antagonistic bacterium, designated SU8, was screened out from a rice-duck farming system and identified as *Pseudomonas aeruginosa* (GenBank: HQ283487). SU8 had a broad antibacterial spectrum, and the strain was not prone to degenerate. The antimicrobial active component of SU8 was identified as phenazine-1-carboxamide (PCN) [27]. Further analysis indicated that PCN has the advantages of a long-lasting period, a rapid control effect, and no environmental pollution, indicating its potential for development as a lead compound. However, the inhibitory effect of PCN against *B*. *cinerea* has rarely been reported. On this basis, the present study was conducted to explore whether PCN has an inhibitory effect against *B*. *cinerea*, to clarify the possible underlying mechanism, and to evaluate whether PCN has potential for the development of new pesticides and for enriching the pool of new agents for the control of *B*. *cinerea*. The results are expected to provide a theoretical basis for the future control of *B*. *cinerea*.

## Materials and Methods

### Reagents and drugs

PCN (93.2%) was isolated from metabolites of the antagonistic bacterium SU8 in our laboratory. Dextrose, agar, beef extract, peptone, and NaCl were purchased from Tiangen, Beijing, China. Ethyl acetate (AR grade), acetone (AR grade), ethanol (AR grade), phosphate buffer, and other common chemical reagents were purchased from Sinopharm Chemical Reagent, Shanghai, China.

### Culture strains

*Botrytis cinerea* was provided by the Laboratory of Plant Pathology, College of Plant Protection, Hunan Agricultural University, China. The antagonistic bacterium SU8 (*Pseudomonas aeruginosa*) was screened from a rice-duck farming ecosystem by the Laboratory of Pesticide Science, Hunan Agricultural University.

### Inhibition and toxicity test of PCN against *B*. *cinerea*

The antagonistic bacterium SU8 was inoculated into beef extract peptone broth medium (NB, beef extract 3.0 g, peptone 10.0 g, NaCl 5.0 g, H_2_O 1000 mL, pH 7.2; the same below) and incubated with shaking (100–120 rpm) at 28–30°C for 72 h (THZ-320 shaker, Shanghai Jinghong Laboratory Instrument Co., Ltd., China; the same below). The culture was filtered using filter paper and then passed through a 0.22-μm bacterium filter to obtain the filtrate. The culture filtrate was mixed with ethyl acetate at a ratio of 1:2 for extraction. The extract was evaporated in a rotary evaporator to form a paste. The obtained paste was mixed with chloroform and petroleum ether at a ratio of 1:4 and then purified using silica gel column chromatography to obtain PCN (93.2% purity). The toxicity of PCN against *B*. *cinerea* was tested using the growth rate method. PCN was weighed and dissolved in a small amount of acetone and then diluted with sterile water to prepare culture filtrates at different concentrations (700, 140, 70, 47, and 35 μg/mL). One millilitre of diluted culture filtrates was mixed with 9 mL of potato dextrose agar medium (PDA, potato 200 g, glucose 17 g, agar 20 g, H_2_O 1000 mL, pH 7.0; the same below) and poured into Petri dishes. *Botrytis cinerea* agar plugs (5 mm diameter) were prepared and placed at the centre of the PDA plates. Carbendazim (97.1% purity,CBM; 15 μg/mL, 30 μg/mL, 60 μg/mL, 120 μg/mL, 240 μg/mL) was used as a control agent. A small amount of acetone mixed with sterile water was used as a blank control. Each treatment was repeated three times. The culture was incubated at 28–30°C for 48 h (SPX-250B incubator, Shanghai Experimental Instrument Factory, China; the same below). Then, the diameters of the colonies were measured to calculate the inhibition rates and EC_50_ [28].

### *In vitro* control effect of PCN against grey mould in strawberry (fruit)

The control effect of PCN was tested according to Huang [29] with appropriate modifications. Strawberry fruits of similar size and free of pests were collected and surface sterilised with 75% alcohol for 3 s. The fruits were then washed three times with sterile water to remove residual alcohol and were placed in a fume hood to dry naturally. Different concentrations (700, 140, 70, 47, and 35 μg/mL) of PCN solutions were smeared on the fruit surface using sterile cotton, and the fruits were then placed in a fume hood to dry naturally. Sterile water was used to inoculate 72-h activated *B*. *cinerea* agar plugs (5 mm diameter) into the fruit base (stipe). CBM (101 μg/mL) was used as a control agent, and sterile water was used as a blank control. The fruits were placed in 15-cm diameter Petri dishes, moisturised and cultured for 48 h. Each treatment was repeated three times, with three fruits per treatment. The plaque diameter was measured using the crossing method, and the control effect was calculated as follows:

$$Control\;effect\;(\%)\;=\;\frac{Disease\;spots\;diameter\;of\;CK-Disease\;spots\;diameter\;of\;treatment}{Disease\;spots\;diameter\;of\;CK-Diameter\;of\;mycelial\;disc}\times 100$$

### Field control effect of PCN against grey mould in strawberry (fruit)

The greenhouse located at the farm of Hunan Agricultural University was chosen as the area for the field control effect test. Serious losses by strawberry gray mould are consistently reported for this farm. During the test process, the greenhouse was maintained at a temperature of 20–25°C and a humidity of 80%; the loose soil contained abundant organic matter and was fertilized evenly. The susceptible variety line Fengxiang was grown in the field under plastic-film-covered cultivation with 20.5-cm row spacing and 16.7-cm line spacing. The PCN was considered the experimental group; a concentration gradient of 35 μg/mL, 47 μg/mL, 70 μg/mL, 140 μg/mL, and 700 μg/mL was used. CBM was the positive control group, and water was the negative control group. There was a total of 7 treatments, with 3 repetitions for each treatment. In the initial stage of gray mould, strawberry leaves were spayed evenly with different test agents and the irrigation volume was controlled at 5 L per 15 m^2^. On the 7th day after drug application, phytotoxicity and damage caused by other non-target organisms were determined. In addition, the extent of the gray mould was surveyed in the field, and the fruit infection rate and control efficiency were calculated according to the national standard of the People's Republic of China GB/T17980.120–2004 [30].

### Effect of PCN on spore and sclerotium germination of *B*. *cinerea*

The effect of PCN on spore germination of *B*. *cinerea* was tested using the slide culture method [29]. PCN was dissolved in a small amount of acetone and diluted with sterile water to prepare different concentrations of working solutions (700, 140, 70, 47, and 35 μg/mL). The obtained spore suspensions were maintained at 4°C (BCD-215KJM refrigerator, Qingdao Haier Group, China) for 2 h, and the spore concentrations were adjusted to 1 × 10^8^ colony-forming units (CFU)/mL. An equal volume of spore suspension was mixed with each PCN dilution and dropped on glass slides. Sterile water was used as the control. Each treatment was repeated three times. The slides were moisturised (12°C) and cultured in Petri dishes for 12 h. Spore germination was examined under a microscope (XSP-8C Nikon optical microscope, Shanghai Precision Instrument Co., Ltd., China).

*B*. *cinerea* was inoculated on PDA plates and cultured at 20–23°C for 3 d. Sclerotia were collected when the cultures turned black and matured. The sclerotia were immersed in a series of PCN solutions (700, 140, 70, 47, and 35 μg/mL) for 48 h. After air-drying, the sclerotia were placed on PDA plates containing the corresponding concentrations of PCN. Each treatment was repeated three times, with 100 measured sclerotia per treatment. The culture was continuously incubated in an incubator at 20–23°C for 48 h. The results of sclerotium germination were compared between treatments, and sclerotium germination rates were calculated.

### Effect of PCN on mycelial growth of *B*. *cinerea*

PCN was dissolved in a small amount of acetone and then diluted with sterile water to prepare a series of working solutions (700, 140, 70, 47, and 35 μg/mL). The PCN dilutions were added to potato dextrose broth (PDB) medium to prepare different medium formulations. Three *B*. *cinerea* agar plugs (5 mm in diameter) were added to each treatment, and each condition was repeated three times. The culture was incubated at 20–23°C with shaking for 4–5 d. The mycelia were collected and washed with distilled water three to four times. After oven drying at 60°C, the mycelia were weighed (AUW320 electronic analytical balance, Shimadzu, Japan), and the inhibition rates of mycelial growth by the agents were calculated as follows:

$$\text{Inhibition rate}\left(\%\right)=\frac{\text{Dry mycelial weight of CK}-\text{Dry mycelial weight of treatment}}{\text{Dry mycelial weight of CK}}\times 100$$

### Effect of PCN on hyphal morphology of *B*. *cinerea*

Hyphal morphology of *B*. *cinerea* was examined following Liu [31] and Soylu [32] with appropriate modifications. Fresh agar blocks (0.8–1 cm) were collected from 96-h-old *B*. *cinerea* cultures from PDA plates of the control (small amount of mixture of acetone and sterile water) and treatment conditions (108.12 μg/mL). The specimens were fixed with 2% glutaraldehyde for 2–4 h, rinsed with 0.1 mol·L phosphate-buffered saline (PBS), and fixed with 1% osmium tetroxide for 1.5 h. After washing with redistilled water until odourless, the specimens were dehydrated with a gradient ethanol series (30%, 50%, 70%, 80%, 90%, 95%, and 100%; 5–10 min each level) followed by isoamyl acetate replacement and conventional critical point drying. Then, the specimens were affixed to the sample tray using a conductive adhesive. Metallic coating was performed in an IB-3 ion coater, and hyphal morphology was examined by SEM (JSM-6380LV, JEOL). Meanwhile, several 1-mm^3^ agar blocks were cut and fixed in 2% glutaraldehyde (pH 7.4) at room temperature for 24 h. The fixed specimens were rinsed with PBS followed by osmium tetroxide fixation, gradient ethanol dehydration, acetone or propylene oxide replacement, and penetration (embedding medium: acetone, 2:1). The previous preparation steps included embedding, aggregation, tissue trimming, ultrathin sectioning, and double-staining with uranyl acetate and lead nitrate. The specimens were observed by TEM (JEM1230, JEOL) and were photographed.

### Action mode of PCN on *B*. *cinerea*

PCN was dissolved and added to PDA medium to prepare 35, 47, and 70 μg/mL PCN-containing plates. *B*. *cinerea* agar plugs were inoculated into the plates and incubated at 20–24°C for 2–3 d. The fungal agar plugs were then transferred to PCN-free PDA plates and incubated for another 2–3 d. If the growth of *B*. *cinerea* was completely inhibited in PCN-containing plates but mycelial growth was observed after transfer, then PCN had fungistatic activity against *B*. *cinerea*; if the growth of *B*. *cinerea* was still completely inhibited after transfer, then PCN had fungicidal activity against *B*. *cinerea*.

### Effect of PCN on the cell membrane permeability of *B*. *cinerea*

The effect of PCN on the cell membrane permeability of *B*. *cinerea* was examined using a conductivity assay [28]. PCN was dissolved and diluted with sterile distilled water to prepare different concentrations (700, 140, 70, 47, and 35 μg/mL) of working solution. The mycelia of 4-day-old *B*. *cinerea* were harvested from PDB medium and processed with a Buchner funnel to drain the surface culture broth and water. The mycelia were washed with distilled water three times, and 2 g of mycelia were weighed into 20 mL of PCN solution series. Sterile distilled water was used as the control. Conductivity was measured at 0, 5, 10, 30, 60, and 120 min at room temperature (DDS-11A digital conductivity meter, Shanghai Precision Instrument Co., Ltd.). The measurement was repeated three times.

### Effect of PCN on *B*. *cinerea* toxin

The effect of PCN on *B*. *cinerea* toxin was based on the methods of Zhu and Moraga [33,34] with appropriate modifications. A *B*. *cinerea* spore suspension (1 mL of 1 × 10^8^ CFU/mL) was cultured in 100 ml Peberdy medium containing PCN (700, 140, 70, 47, or 35 μg/mL); non-PCN was used as the control and each treatment was replicated 3 times. After stilling the culture for 18–20 d at 22°C, the fermentation broth was centrifuged at 10,000 rpm for 5 min to obtain a fermented filtrate of *B*. *cinerea*. The pathotoxin of *B*. *cinerea* was extracted with the same volume of chloroform and its OD_269_ was measured using a UVVIS spectrophotometer.

### Effects of PCN on cell wall-degrading enzyme activities of *B*. *cinerea*

Activated *B*. *cinerea* agar plugs were inoculated into PDB medium, followed by the addition of prepared PCN solutions (5, 15, 20, 35, 47, 70, 140 and 700 μg/mL). The control was prepared without the addition of the PCN solutions. After 48 h of treatment, wet mycelia were collected after filtration, and 2 g of each sample was accurately weighed from each treatment. Pectin methylgalacturonase (PΜG), polygalacturonase (PG), β-glucosidase (BG), and cellulase (Cx) activity assays were performed according to the method of Chen and Li [35,36]. The activity of PMG and PG were defined as the amount of PMG or PG required to catalyze substrate to form 1μmol reducing sugar in one hour. The activity of BG and Cx were defined as the amount of BG or Cx required to catalyze substrate to form 1μmol galacturonic acid in one hour.

### Statistical analysis

The experimental data were subjected to analysis of variance using SPSS11.5 software. Significant treatment differences were tested using Duncan’s new multiple range method.

## Results

### Inhibition and indoor toxicity of PCN against *B*. *cinerea*

**Fig 1:**
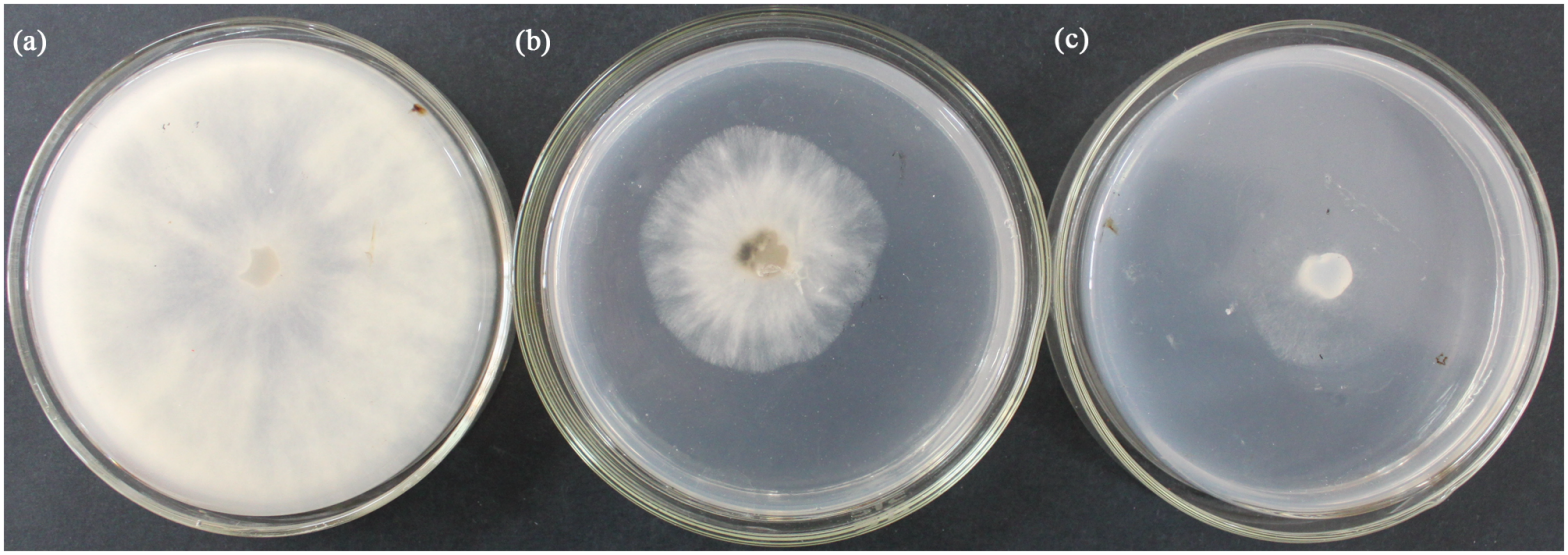
Inhibitory effect of PCN against *B*. *cinerea* (plate growth rate method). Note: (a): CK; (b): CBM (101.39 μg/mL); (c): PCN (108.12 μg/mL).

As shown in Fig 1, PCN showed inhibitory effects against *B*. *cinerea*, which suggest that the natural compound can be used to enrich the agent pool for controlling strawberry grey mould in the future.

Further analysis (Table 1) revealed that high concentrations of PCN had better inhibitory effects against *B*. *cinerea*, with the highest inhibition rate of 68.54%. The 50% effective concentration (EC_50_) of PCN was 108.12 μg/mL, which slightly exceeded that of CBM (101.39 μg/mL), indicating the lower toxicity of PCN compared with the control agent. The above result indicates that the natural compound can be further studied by modifying its structure, adding additives, or increasing the dosage to improve its fungicidal activity before its applications are extended in the field.

**Table 1:** Toxicity and inhibition of PCN against *B*. *cinerea*.

Treatment	Concentration (μg/mL)	Inhibition rate (%)	Regression equation	EC_50_(μg/mL)
**PCN**	35	24.67±2.45^d^	y = 0.8337x+3.3043	108.12
	47	36.39±3.64^c^		
	70	50.78±2.07^b^		
	140	66.75±3.67^a^		
	700	68.54±2.85^a^		
**CBM**	15	12.55±1.26^e^	y = 1.4738x+2.0437	101.39
	30	17.27±1.73^d^		
	60	39.15±3.92^c^		
	120	57.11±2.71^b^		
	240	69.46±1.93^a^		

Note: Mean values ± SD followed by different letters indicate significantly different scores in the same phase, according to Duncan’s multiple range tests at the P = 0.05 level.

### *In vitro* control effect of PCN against grey mould in strawberry (fruit)

The PCN concentration was directly proportional to its control effect (Table 2). At a concentration of 700 μg/mL, the control effect of PCN reached 75.32%, that is, 2.65-fold higher than the control effect of the lowest concentration treatment. The control effect of PCN was equivalent to that of CBM, with no significant difference. Furthermore, in the concentration range of 35–700 μg/mL, PCN did not affect the normal growth of strawberry, and no plant disease phenomena were observed. These results suggest that PCN can not only address the problem of low control effect against strawberry grey mould but can also serve as an alternative for replacing the existing pesticides in production that are associated with pathogen resistance. Thus, the new compound PCN has broad development prospects.

**Table 2:** Control effect of PCN against *B*. *cinerea*.

Treatment	Concentration (μg/mL)	Control effect (%)
**PCN**	35	28.39±2.84^e^
	47	31.38±3.14^d^
	70	46.61±1.66^c^
	140	62.41±2.24^b^
	700	75.32±1.53^a^
**CBM**	101	74.99±3.50^a^

Note: Mean values ± SD followed by different letters indicate significantly different scores in the same phase, according to Duncan’s multiple range tests at the P = 0.05 level.

### Field control effect of PCN against grey mould in strawberry (fruit)

There was positive correlation between the PCN concentrations and field control efficacy (Table 3). The efficiency on *B*. *cinerea* exceeded 72.31% when concentration of PCN reached 700 μg/mL, which was 1.02 times higher than that measured in the control group of CBM. The groups exhibited equivalent levels of control with respect to the diseased fruit rate; however, comparisons with lower PCN concentrations were significant. The fruit infection rate and the disease-control efficiency were maintained between 43.6–79.33% and 10.39–50.72%, respectively, when the PCN concentration was within the range of 35–140 μg/mL, which indicated that PCN had a greater affect at higher concentration. Neither the recommended nor the higher rates of PCN caused phytotoxicity with respect to blade shape, leaf color, fruit and other non-target organisms.

**Table 3:** Control effect of PCN against *B*. *cinerea* in field.

Treatment	Concentration (μg/mL)	Control effect (%)
**PCN**	35	28.39±2.84^e^
	47	31.38±3.14^d^
	70	46.61±1.66^c^
	140	62.41±2.24^b^
	700	75.32±1.53^a^
**CBM**	700	74.99±3.50^a^

Note: Mean values ± SD followed by different letters indicate significantly different scores in the same phase, according to Duncan’s multiple range tests at the P = 0.05 level.

### Effect of PCN on spore germination

Spores are an important basis for species identification and a primary infection source in the occurrence of plant diseases. The number of spores is a prerequisite that determines whether a plant disease can occur and prevail. Therefore, testing the effect of a potential control agent on spore germination can directly determine whether the plant disease can occur and prevail. PCN had an inhibitory effect against *B*. *cinerea* spore germination (Table 4). Higher concentrations of PCN resulted in lower spore germination rates, which corresponded to a higher relative inhibition rate of spore germination. At PCN concentrations of 35–70 μg/mL, the spore germination rate of *B*. *cinerea* ranged from 77–88%, and the relative inhibition rate of spore germination ranged from 10.45–21.62%. When the PCN concentration was increased to 140 μg/mL, the spore germination rate of *B*. *cinerea* was 65%, and the relative inhibition rate of spore germination was 33.44%. At the highest PCN concentration of 700 μg/mL, the spore germination rate of *B*. *cinerea* was only 22%, while relative spore germination exhibited inhibition rates as high as 77.03%, showing strong inhibition.

**Table 4:** Effect of PCN against *B*. *cinerea* spore germination.

Concentration (μg/mL)	Spore germination rate (%)	Spore germination relative inhibition rate (%)
**35**	88.35±0.58^b^	10.45±1.05^d^
**47**	78.82±1.01^c^	20.93±2.10^c^
**70**	77.01±0.57^c^	21.62±2.17^c^
**140**	65.22±0.58^d^	33.44±3.34^b^
**700**	22.18±0.58^e^	77.03±2.71^a^
**CK**	99.03±0.56^a^	

Note: Mean values ± SD followed by different letters indicate significantly different scores in the same phase, according to Duncan’s multiple range tests at the P = 0.05 level.

### Effect of PCN on sclerotium germination of *B*. *cinerea*

Sclerotia, the resting bodies of *B*. *cinerea*, are formed to resist adverse environmental conditions. Under suitable environmental conditions, sclerotia can germinate and re-infect the host. Thus, sclerotia are an important primary infection source causing strawberry grey mould. *B*. *cinerea* sclerotium germination was affected by PCN treatment (Table 5). The sclerotium germination rate was higher with lower concentrations of PCN, and the rates decreased with higher concentrations of PCN. At a PCN concentration of 35 μg/mL, the sclerotium germination rate reached 95%, while the relative inhibition rate of sclerotium germination was only 7.09%. When the concentration of PCN was increased to 700 μg/mL, the sclerotium germination rate was as low as 17%, while the inhibition rate of sclerotium germination was as high as 82.11%. These results indicate that PCN can inhibit *B*. *cinerea* sclerotium germination, thereby reducing the primary infection sources of the fungal pathogen and achieving the goal of disease control. Furthermore, the germination of untreated sclerotia after their inoculation onto culture plates was unaffected. Collectively, these results demonstrate that PCN can inhibit both spore and sclerotium germination of *B*. *cinerea*.

**Table 5:** Effect of PCN on *B*. *cinerea* sclerotium germination.

Concentration (μg/mL)	Average germination rate (%)	Sclerotium germination relative inhibition (%)
**35**	91.11±0.57^b^	7.09±0.01^d^
**47**	82.32±0.58^c^	16.54±0.02^c^
**70**	64.33±1.01^d^	35.12±0.02^b^
**140**	64.25±1.03^d^	35.13±0.01^b^
**700**	17.05±1.53^e^	82.11±0.02^a^
**CK**	99.12±0.58^a^	

Note: Mean values ± SD followed by different letters indicate significantly different scores in the same phase, according to Duncan’s multiple range tests at the P = 0.05 level.

### Effect of PCN on *B*. *cinerea* mycelial growth

PCN significantly inhibited *B*. *cinerea* mycelial growth (Table 6). The higher the concentration of PCN, the more obvious the inhibition of mycelial growth, the lower the mycelia weight, and the stronger the inhibitory effect on mycelial growth. When the concentration of PCN was 35–47 μg/mL, the inhibition rate of mycelial growth was lowest, ranging from 6.37–7.44%. As the concentration of PCN increased to 70–140 μg/mL, the inhibition rate of mycelial growth ranged from 53.34–74.38%. At the highest PCN concentration of 700 μg/mL, the inhibition rate of mycelial growth was as high as 87.32%.

**Table 6:** Effect of PCN on *B*. *cinerea* mycelia growth.

Concentration (μg/mL)	Weight (g)	Inhibition rate (%)
**700**	0.26±0.01^f^	87.32±0.01^a^
**140**	0.53±0.02^e^	74.38±0.02^b^
**70**	0.95±0.03^d^	53.34±0.01^c^
**47**	1.91±0.01^b^	6.37±0.01^e^
**35**	1.89±0.02^c^	7.44±0.02^d^
**CK**	2.05±0.01^a^	

Note: Mean values ± SD followed by different letters indicate significantly different scores in the same phase, according to Duncan’s multiple range tests at the P = 0.05 level.

### Action mode of PCN on *B*. *cinerea*

A small amount of mycelia grew from *B*. *cinerea* agar plugs on the 35 μg/mL PCN-containing plates, whereas no mycelia grew from fungal agar plugs on the 47 μg/mL PCN-containing plates. The fungal agar plugs were transferred to PCN-free plates and incubated for another 5–7 d. The mycelia grew into colonies similar in size to those on the control plates, suggesting that low concentrations (35–47 μg/mL) of PCN only had fungistatic activity against *B*. *cinerea*. However, when the fungal agar plugs (showing no hyphal growth) treated with 70 μg/mL or higher concentrations of PCN were transferred to PCN-free plates, no growth of *B*. *cinerea* was observed, indicating that 70 μg/mL or higher concentrations of PCN had fungicidal activity against *B*. *cinerea*.

### Effect of PCN on *B*. *cinerea* hyphal morphology

Colony observation of *B*. *cinerea* revealed that this fungus extended radially on the PDA plates. Colonies grown on PCN-containing plates had sparse and twisted mycelia, indicating slow growth. Colonies grown on PCN-free plates were thicker; the mycelia grew closely to the medium and appeared relatively upright, indicating vigorous growth.

**Fig 2:**
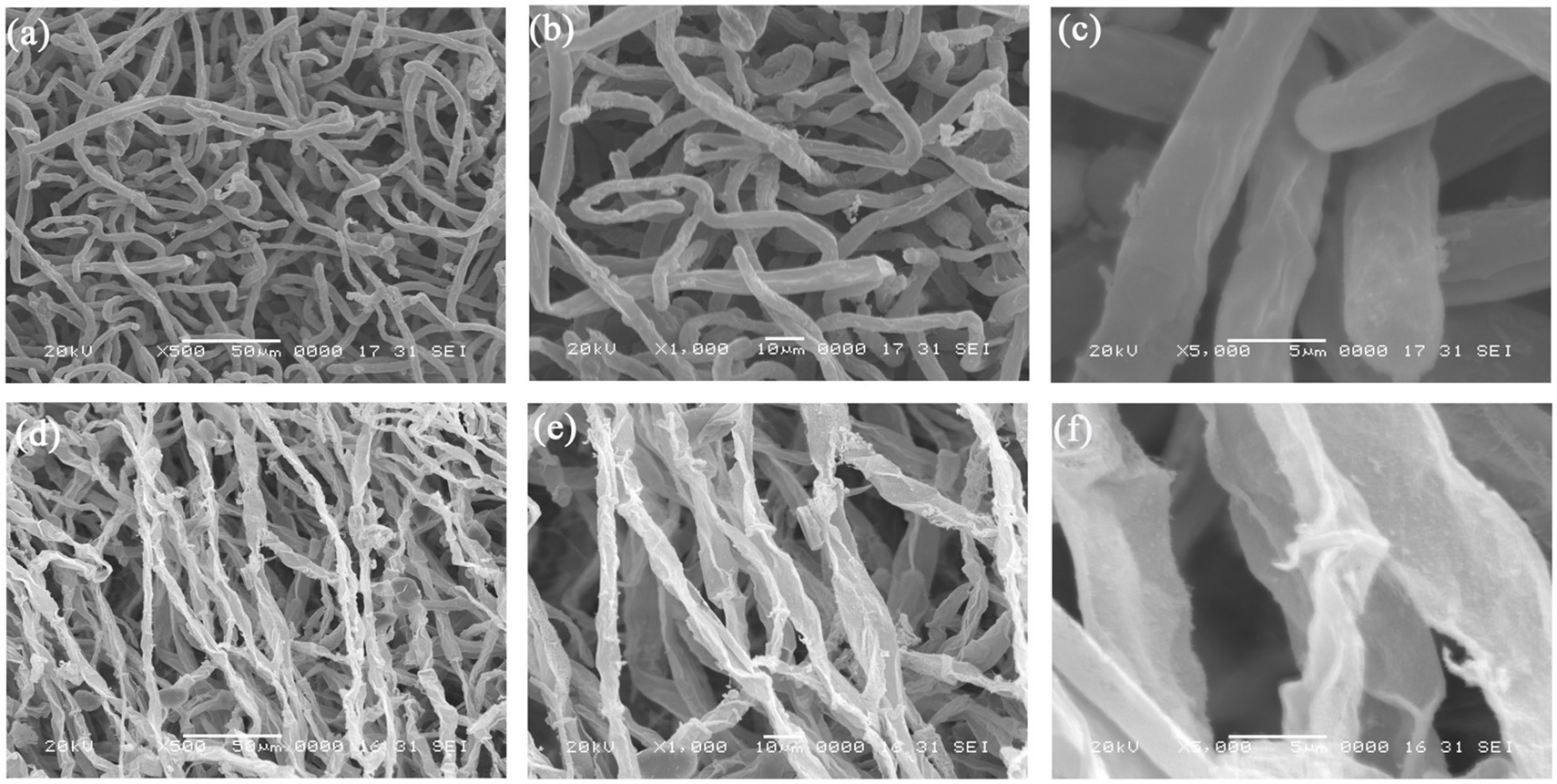
Scanning electron microscopy of the morphology of mycelia exposed to PCN. (a, b, and c) Healthy mycelia in control Petri plates. (d, e, and f) Effects of PCN at 108.12 μg/mL on hyphal morphology. (a) 500x, (b) 1000x and (c) 5000x: control mycelia; (d) 500x, (e) 1000x and (f) 5000x: treated mycelia.

SEM observations revealed that after 48 h of PCN treatment, the mycelia were loose, locally twisted and folded, with exudation of contents; the mycelia were withered and twisted, with edge burrs, deformations, ruptures and a sheet-like structure (Fig 2d–2f). In contrast, untreated mycelia were slender and uniform, with a smooth surface and an intact structure (Fig 2a–2c).

**Fig 3:**
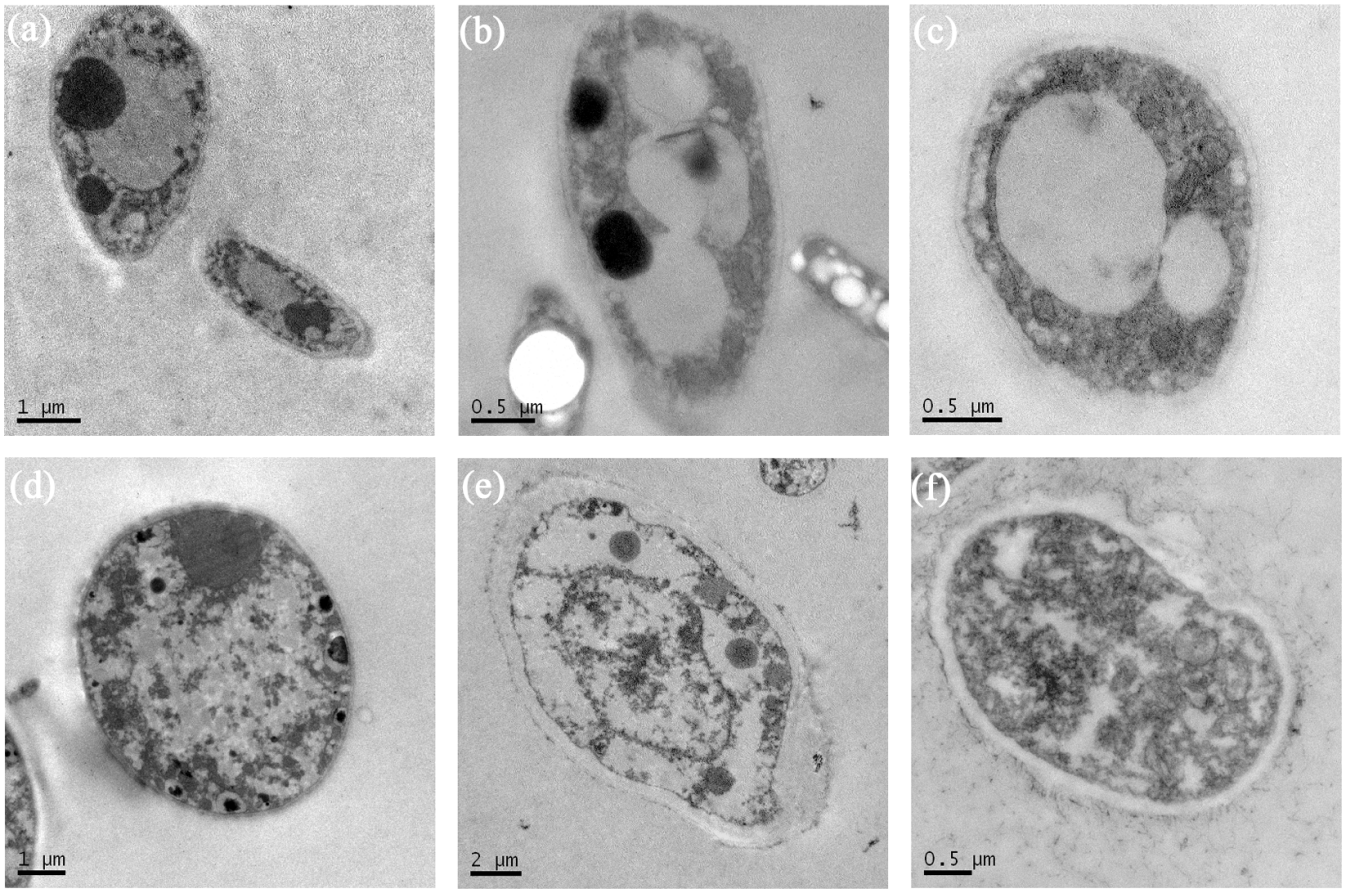
Transmission electron microscopy of hyphal ultrastructures in mycelia exposed to PCN. (a, b and c) Healthy mycelia in control Petri plates. (d, e and f) Effects of PCN concentration at 108.12 μg/mL on hyphal ultrastructure. (a) 5000x, (b) 10000x, and (c) 50000x, control mycelia; (d) 5000x, (e) 10000x and (f) 50000x: treated mycelia.

TEM observations revealed that after 48 h of PCN treatment, the structures of cell nuclei were unclear, and the vacuoles had ruptured. Additionally, various organelles presented disordered structures, and there were substantial non-membrane transparent inclusions. Furthermore, the cells were plasmolysed, with the cell wall collapsed in some cases, and the hyphal tissue was essentially necrotic (Fig 3d–3f). In untreated hyphal cells, organelles exhibited intact and dense structures, with uniform cell matrices and no extracellular extravasation; the mitochondria were uniform, and the nuclei were intact (Fig 3a–3c).

### Effect of PCN on *B*. *cinerea* cell membrane permeability

**Fig 4:**
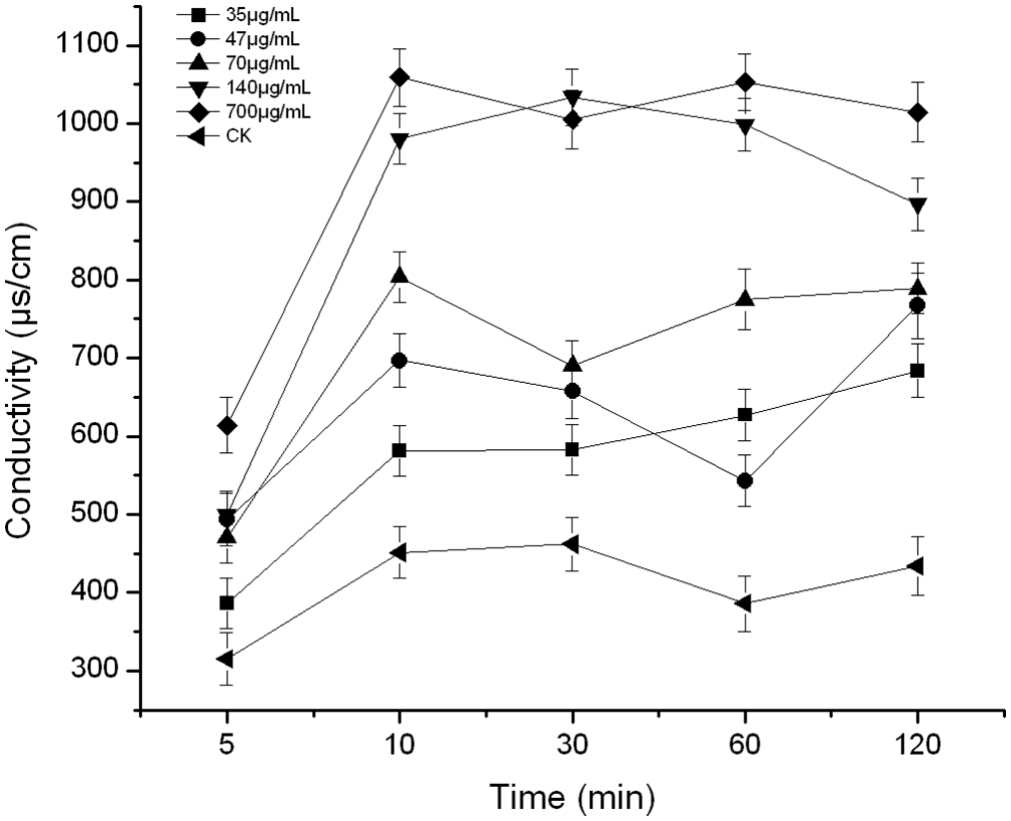
Effect of PCN on *B*. *cinerea* membrane permeability. Note: Mean values ± SD followed by different letters indicate significantly different scores in the same phase, according to Duncan’s multiple range tests at the P = 0.05 level.

Conductivity changes can reflect alterations in cell membrane permeability. As shown in Fig 4, the cell membrane conductivity values of PCN-treated *B*. *cinerea* were consistently higher than those of the control. The conductivity gradually increased with increasing PCN concentration. When the concentration of PCN was in the range of 35–140 μg/mL, there were no significant conductivity changes compared with the control. However, when the concentration of PCN increased to 700 μg/mL, the conductivity significantly increased after 60 min compared with the control. These results indicated that high concentrations of PCN can increase cell membrane permeability, resulting in electrolyte leakage.

### Effect of PCN on *B*. *cinerea* toxin

**Fig 5:**
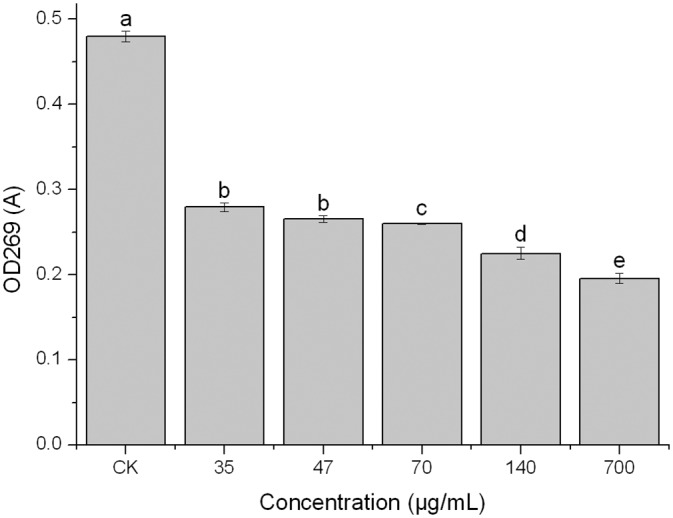
Effects of PCN on *B*. *cinerea* toxin. Note: Mean values ± SD followed by different letters indicate significantly different scores in the same phase, according to Duncan’s multiple range tests at the P = 0.05 level.

Plant pathogenic mycotoxins are one of the reasons for plant disease. Colmenares’ research suggested that the mycotoxin of *B*. *cinerea* could change leaf color, cause cell disruption, promote invasion and expansion processes, and lead to synergistic pathopoiesis with exoenzymes [37]. It is evident in Fig 5 that the inhibitory effect of PCN on the production of *B*. *cinerea* toxin increased with an increase in the concentration of PCN, i.e., the lower the level of mycotoxin produced by *B*. *cinerea*. A PCN concentration of 35 μg/mL showed the lowest inhibitory effect while 700 μg/mL of PCN was optimal, i.e., the mycotoxin level was approximately 0.41 of the value recorded for the control. Variance analysis indicated significant differences between the various PCN concentration treatments. These results indicated that PCN can delay the infection of strawberry gray mould by inhibiting the mycotoxin of *B*. *cinerea*.

### Effect of PCN on enzyme activity of *B*. *cinerea*

Pectinases are a class of enzymes that break down pectin. These types of enzymes can break down the complex structure of pectin into galacturonic acid and other small molecules. PMG and PG are pectinases produced in the pathogenic process of *B*. *cinerea* and have a role in degrading pectin in strawberry [38]. BG and Cx are cellulases generated in the pathogenic process of *B*. *cinerea* and play a role in degrading the fibrous tissue of the fruit [35].

**Fig 6:**
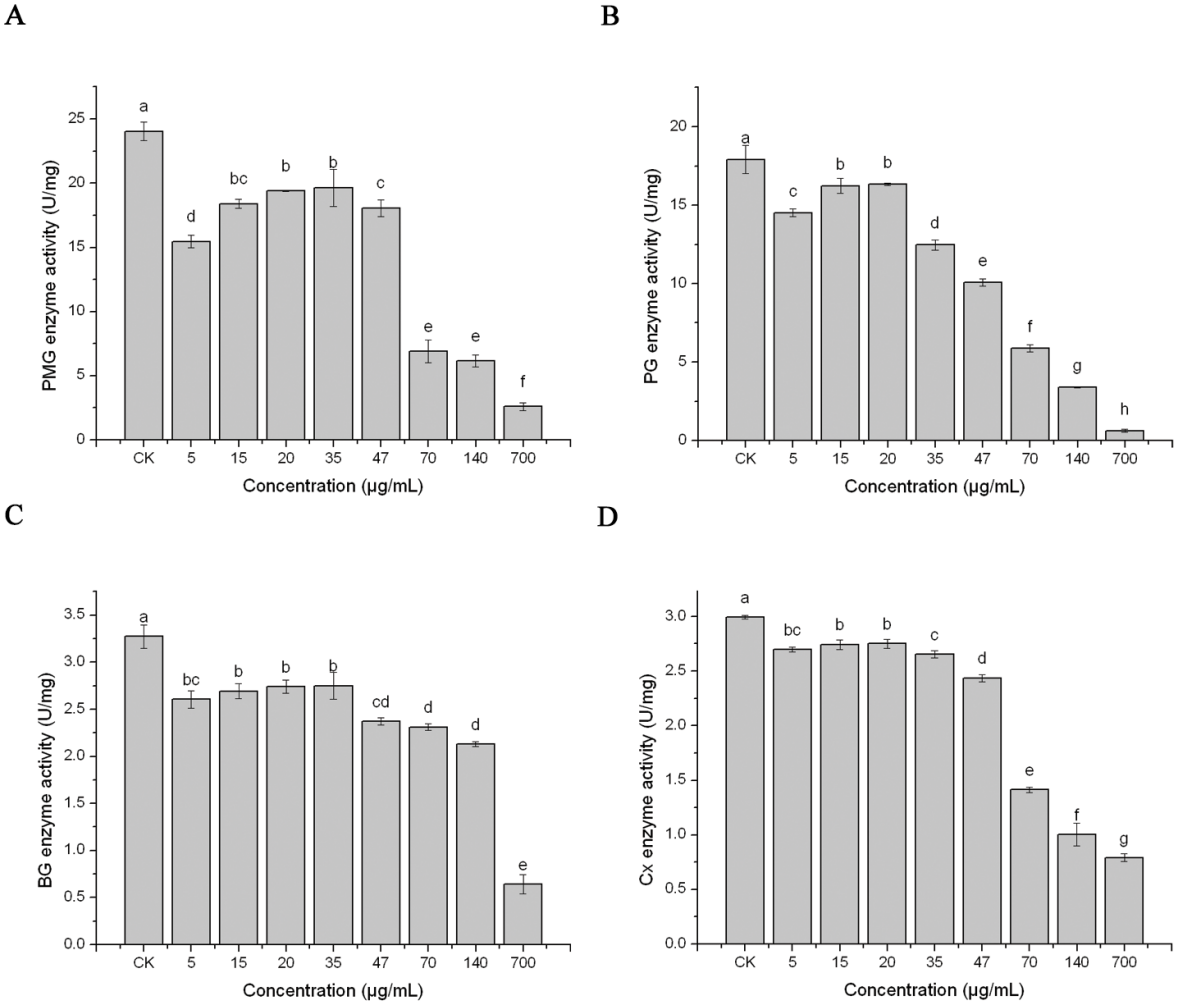
Effects of PCN on *B*. *cinerea* cell wall-degrading enzyme (PMG PG BG and Cx) activities. Note: Mean values ± SD followed by different letters indicate significantly different scores in the same phase, according to Duncan’s multiple range tests at the P = 0.05 level.

As shown in Fig 6, after treatment with PCN (at concentrations of 5–700 μg/mL), the enzyme activities of PMG, PG, BG, and Cx were reduced compared with CK. The patterns exhibited by the four enzymes corresponded to the concentration of PCN. At low PCN concentrations, the inhibition action on the four enzymes was weak, and at a PCN concentration ranging from 5–35 μg/mL, the PMG and BG activities increased with an increase in the PCN concentration. Similar results were observed for the PG and Cx activities at a PCN concentration of 5–20 μg/mL. However, with a further increase in the PCN concentration, the activities of PMG, PG, BG, and Cx were obviously restricted, and the lowest activities of PMG, PG, BG, and Cx reached 0.3 U/mg, 0.62 U/mg, 0.64 U/mg, and 0.79 U/mg, respectively, after treatment with 700 μg/mL PCN.

## Discussion

This study demonstrated that PCN (EC_50_ of 108.12 μg/mL) was inhibitory against *B*. *cinerea*; the toxicity of this natural compound was slightly lower than that of CBM, the control agent. *In vitro* test results showed that the control effect of PCN against grey mould in strawberry fruit reached 75.32%, which is equivalent to that of CBM (74.99%). Because *B*. *cinerea* can propagate rapidly and with great variability, this pathogen gains resistance to chemical agents commonly used in production (e.g., procymidone, azoxystrobin, CBM, iprodione, pyrimethanil, and fludioxonil), resulting in declines in the control effect [39, 40]. It is well known that one of the means to address drug resistance is the screening of new drugs. This study screened PCN, a new agent that has not previously been used in strawberry production. To a certain extent, this study alleviates the problem regarding *B*. *cinerea* drug resistance. Meanwhile, the results indicate that PCN has the potential to aid in the development of new control agents against strawberry grey mould. However, only *in vitro* experiments were performed in this study, and these types of experiments differ from field experiments.

The field experiment showed that PCN had a good ability to control the disease; while the indoor protection effect was slightly higher than the field efficacy, the field control effect was 72.31% higher than that recorded for the control carbendazol. Many factors such as ultraviolet radiation, temperature, humidity, pH, microorganisms and so on consistently impacted the efficacy of PCN in the field experiment. Some problems remain that require further discussion and determination such as the controlling mode of PCN (protection, therapeutic effects or eradicant action), the best period for prevention, the environmental behavior of PCN and the control efficiency of PCN in different climatic zones.

Previous research has confirmed that another method to develop new pesticides is to screen agents with different control mechanisms, which can be effective solutions to address *B*. *cinerea* drug resistance or tolerance. On this basis, we further studied the control mechanism of PCN on *B*. *cinerea*. The results showed that PCN reduced the primary infection sources of *B*. *cinerea* by inhibiting spore and sclerotium germination; the inhibition rates ranged from 22–88% and 17–91%, respectively. Additionally, strong inhibition of mycelial growth [35] was observed with PCN, with an inhibition rate that ranged from 6.37–87.32%. This result indicates that PCN can inhibit *B*. *cinerea* mycelial growth, reduce its biomass, and decrease its vitality. Low PCN concentrations had fungistatic effects, while high PCN concentrations had fungicidal effects against *B*. *cinerea*. The analysis of cell membrane permeability revealed that low PCN concentrations had no effect on cell membrane permeability of *B*. *cinerea*, while high PCN concentrations resulted in significant leakage. A possible mechanism is that high concentrations of chemical agents lead to changes in cell membrane permeability, thereby causing conductivity increases and further resulting in the leakage of the intracellular protoplasm [41].

This study indicated that PCN had certain effects in delaying fungal infection of the host cell wall by inhibiting the production of *B*. *cinerea* toxins. Some issues remain to be resolved, such as the influence of PCN on toxin-producing efficiency, how the toxin works and how to develop PCN as a toxin inhibitor.

Pectinase and cellulase are pathogenic enzymes of *B*. *cinerea* in host plant infections. The activity levels of these enzymes reflect the strength of fungal pathogenicity. The study showed that PCN had an inhibitory effect on the activity of pectinase (PMG, PG) and cellulase (BG, Cx), but at certain low concentrations of PCN, the inhibition effect on the four enzymes was weak and enzyme activities increased with an increase in the concentration of PCN. It is assumed that this phenomenon occurred because low concentrations of PCN may play a role in maintaining the active conformation of the enzyme, while PCN at high concentrations may bind to other amino acid residues of enzymes, which could change their conformation and interfere with enzyme activity [42,43]. However, the actual cause remains to be determined.

These results are completely different from the control mechanisms of other commonly used chemical reagents. For example, CBM affects cell division by inhibiting spindle formation during mitosis; procymidone interferes with pathogen growth by inhibiting triglyceride synthesis; fludioxonil prevents mycelial growth by inhibiting the transfer of glucose phosphorylation; and iprodione controls pathogen growth by inhibiting protein kinase activity [44]. Although our research group has obtained considerable research results, for the sake of brevity, this study only discussed the apparent biological mechanism of PCN on *B*. *cinerea*. The action sites of PCN were detected in the cell wall and the cell membrane. However, the molecular mechanisms of PCN remain to be explored, particularly regarding whether PCN can inhibit protein synthesis, affect DNA replication, or influence intracellular mitochondrial respiration [45]. Moreover, the extraction and dissolution (to constant volume) processes of active PCN require heating, which may result in the loss of certain volatile antimicrobial components. Future trials will need to modify the preparation methods to improve the antimicrobial activity of PCN. In conclusion, PCN has the advantages of high efficiency, low toxicity, low production costs, and a unique mechanism for controlling *B*. *cinerea*. This natural compound can be further developed into a biological agent to control grey mould in strawberry.
